# Early assessment of acute coronary syndromes in the emergency department: the potential diagnostic value of circulating microRNAs

**DOI:** 10.1002/emmm.201201749

**Published:** 2012-10-01

**Authors:** Martinus I F J Oerlemans, Arend Mosterd, Marieke S Dekker, Evelyn A de Vrey, Alain van Mil, Gerard Pasterkamp, Pieter A Doevendans, Arno W Hoes, Joost P G Sluijter

**Affiliations:** 1 Department of Cardiology, University Medical Center UtrechtThe Netherlands; 2 Julius Center for Health Sciences and Primary Care, University Medical CenterUtrecht, The Netherlands; 3 Department of Cardiology, Meander Medical CenterAmersfoort, The Netherlands; 4 Department of Cardiology, Isala ClinicsZwolle, The Netherlands; 5 Interuniversity Cardiology Institute Netherlands (ICIN)Utrecht, The Netherlands

**Keywords:** acute coronary syndrome, circulating microRNA, miR-1, miR-21, miR-499

## Abstract

Previous studies investigating the role of circulating microRNAs in acute coronary syndrome (ACS) were based on small patient numbers, performed no comparison with established markers of cardiac injury and did not have appropriate controls. We determined the potential diagnostic value of circulating microRNAs as novel early biomarkers in 332 suspected ACS patients on presentation to the emergency department (ED) in a prospective single-centre study including cardiac miRNAs (miR-1, -208a and -499), miR-21 and miR-146a. Levels of all miRs studied were significantly increased in 106 patients diagnosed with ACS, even in patients with initially negative high-sensitive (hs) troponin or symptom onset <3 h. MiR-1, miR-499 and miR-21 significantly increased the diagnostic value in all suspected ACS patients when added to hs-troponin T (AUC 0.90). These three miRs were strong predictors of ACS independent of clinical co-variates including patient history and cardiovascular risk factors. Interestingly, the combination of these three miRs resulted in a significantly higher AUC of 0.94 than hs-troponin T (0.89). Circulating microRNAs hold great potential as novel early biomarkers for the management of suspected ACS patients.

## INTRODUCTION

Acute coronary syndrome (ACS) remains one of the leading causes of morbidity and mortality in the Western world. Early diagnosis of ACS is essential because of improvement in prognosis following timely interventions. Currently, the diagnosis of ACS is based on elevation of (high-sensitive) cardiac troponin I or T (cTnI or cTnT), in the context of clinical and electrocardiographic (electrocardiogram, ECG) findings (Anderson et al, 2011; Thygesen et al, 2007). Unfortunately, these biomarkers are not consistently elevated within the first hours after symptom onset, requiring repetitive measurements and hindering early diagnosis (Dekker et al, 2010). In the setting of typical symptoms and ST segment elevation on the ECG, the diagnosis and subsequent management is straightforward (Kushner et al, 2009). In daily clinical practice, however, this scenario only pertains to a minority of patients presenting to the emergency department (ED) with symptoms suggestive of an ACS, making the early diagnosis of ACS a challenge and new diagnostic markers to improve early recognition of ACS are required.

MicroRNAs (miRNAs) are short, non-coding small RNAs that regulate the expression of proteins by translational repression or degradation of messenger RNAs (mRNAs; Lewis et al, 2003). Over the last few years, it has been established that miRNAs play a crucial role in cardiac development and homeostasis and that miRNA expression is altered in the diseased heart (Sluijter et al, 2010; Thum et al, 2007; van Rooij et al, 2006). Interestingly, miRNAs were also found to be present in human serum and plasma and altered expression profiles were observed in cancer and other diseases like diabetes (Chen et al, 2008; Mitchell et al, 2008; Zampetaki et al, 2010). This led to the hypothesis that miRNAs might be released upon cardiac injury and that the detection of cell-free miRNAs – including cardiac-related miR-1, miR-499 and miR-208a – could be used for the diagnosis of ACS (Adachi et al, 2010; Ai et al, 2010; Cheng et al, 2010; Corsten et al, 2010; D'Alessandra et al, 2010; Gidlof et al, 2011; Ji et al, 2009; Kuwabara et al, 2011; Olivieri et al, 2012; Wang et al, 2010a, b; Widera et al, 2011). Previous studies investigating miRNAs for detection of cardiac injury have been relatively small (mostly including <100 patients) and were not carried out in the clinically relevant patient population (*i.e.* unselected patients suspected of ACS presenting to the ED), but typically included patients already known to have coronary artery disease [*e.g.* those undergoing primary percutaneous coronary intervention (PCI)] and compared these with healthy controls (Corsten et al, 2010; D'Alessandra et al, 2010; De Rosa et al, 2011; Wang et al, 2010a, b). In addition, several studies used samples taken at the time of reperfusion to determine miRNAs, rather than samples taken upon initial presentation to the ED, when diagnostic uncertainty is most evident.

Our aim was to determine the diagnostic value of circulating miRNAs, in particular cardiac-related miR-1, miR-499 and miR-208a, and stress-related miR-21 and miR-146a (Dong et al, 2009; Horie et al, 2010; van Rooij et al, 2008) in the early assessment of suspected ACS patients presenting at the ED. In addition, we determined the diagnostic value of miRNAs in subgroups of suspected ACS patients with initially negative high-sensitive (hs-) troponin levels and in those presenting within 3 h of symptom onset since in these patients, the diagnostic accuracy of available biomarkers is limited.

## RESULTS

### Patient characteristics

The median onset of chest pain in the 332 suspected ACS patients was 3.2 h [interquartile range (IQR) 1.8–8.0] prior to presentation (Table 1). ACS was diagnosed in 106 (31.2%) patients, consisting of UA (*n* = 24, 22.6%) and NSTEMI (*n* = 82, 77.4%). Of the ACS-patients, 21.7% (23/106) had a hs-troponin level <0.14 pg/ml at initial presentation with a median hs-troponin of 7.25 (IQR 3.1–11.3) whereas in the hs-troponin >14 pg/ml this was 41.2 (IQR 18.7–108.2).

**Table 1:** Patient characteristics of 332 patients with chest pain

Characteristic	Non-ACS	ACS	All	*p*-value
*N* (%)	226 (68.1)	106 (31.2)	332 (100)	
Age (yrs)	60.2 ± 14.3	68.7 ± 12.6	62.9 ± 14.4	0.00
Male sex (% male)	120 (53.1)	70 (66.0)	190 (57.2)	0.03
Onset of chest pain, median (IQR)	3.2 (1.9–9.0)	3.1 (1.6–8.0)	3.2 (1.8–8.0)	0.64
Risk factors				
Current smoker	62 (27.7)	24 (22.9)	86 (26.1)	0.42
Former smoker	71 (31.8)	32 (30.8)	103 (31.5)	0.90
Hypertension	90 (39.8)	61 (58.7)	151 (45.8)	0.02
Hypercholesterolemia	65 (28.9)	44 (41.9)	109 (33.0)	0.02
Diabetes mellitus	31 (13.8)	21 (20.0)	52 (15.8)	0.19
Body mass index (kg/m^2^)	26.4 ± 6.1	26.0 ± 5.1	26.3 ± 5.8	0.34
Parental CVD	89 (39.7)	44 (42.7)	133 (40.7)	0.63
History of CVD	97 (42.9)	62 (58.5)	159 (47.9)	0.01
Cardiac Troponin I (µg/L)	0.02 (0.01–0.03)	0.07 (0.04–0.21)	0.03 (0.02–0.05)	0.00
Cardiac hs-Troponin T (pg/ml)	3.3 (1.1–8.5)	23.7 (10.6–80.8)	6.0 (2.2–18.7)	0.00

Data presented as mean ± SD for age, body mass index and as median (25th to 75th IQR) for onset of chest pain and cardiac troponin levels. All other variables are presented as n (%); ACS, acute coronary syndrome; CVD, cardiovascular disease; Mann–Whitney (continuous variables) or *χ*^2^ test (categorical variables), ACS (*n* = 106) *versus* non-ACS (*n* = 226).

In 226 (68.1%) patients an ACS could be ruled out; in the non-ACS population, the following diagnoses were established by the expert-panel: stable angina (*n* = 39), rhythm disorders (*n* = 9), heart failure (*n* = 3), pericarditis (*n* = 2), other cardiac diagnosis (unlisted, *n* = 12) and non-cardiac chest pain (*n* = 161).

### Circulating miRNA levels in ACS patients

**Figure 1:**
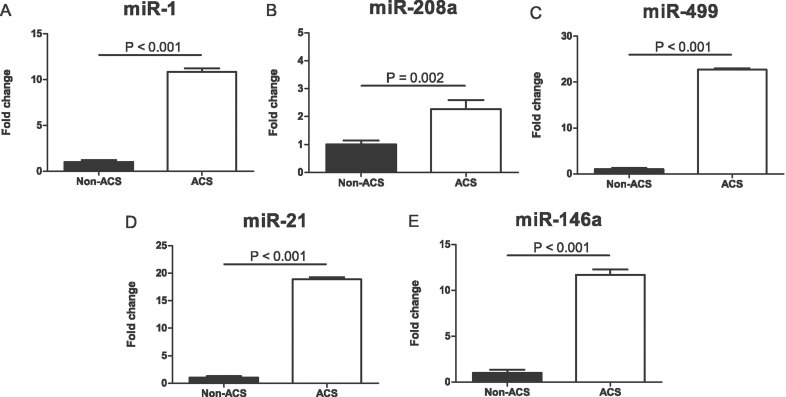
Expression levels of circulating miRNAs in serum of ACS and non-ACS patients Data are presented as mean ± SEM, *p*-values *versus* non-ACS patients, Mann–Whitney test. **A–C.** Cardiac-specific miR-1, miR-208a and miR-499 were significantly increased in ACS patients.**D,E.** Both levels of miR-21 (**D**) and miR-146a (**E**) were markedly elevated in ACS patients compared to non-ACS patients.

Compared to the non-ACS population, circulating levels of all cardiac-expressed miRNAs studied (miR-1, miR-499 and miR-208a) were higher in patients with ACS (Fig 1A–C). Furthermore, circulating levels of miR-21 (18.9-fold) and miR-146a (11.7-fold) were markedly elevated in ACS patients as well (*p* < 0.001; Fig 1D and E). None of the circulating miRNAs were significantly associated with age, gender or other relevant clinical co-variates (Supporting Information Table S1). Furthermore, heparin treatment or platelet inhibition (aspirin, clopidogrel) at presentation was not significantly correlated with any of the circulating miRs as depicted in Supporting Information Table S2.

### Expression pattern of circulating miRNAs in UA and NSTEMI patients

**Figure 2:**
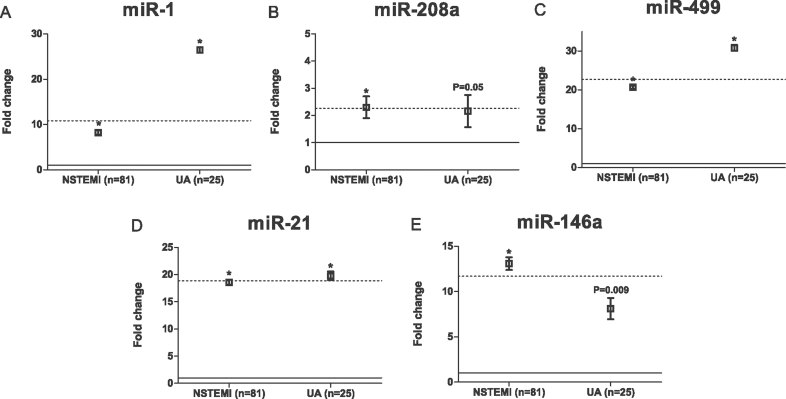
Expression pattern of circulating miRNAs in STEMI and UA patients Circulating miRNAs displayed different expression levels within the ACS population. Dotted lines represent fold increase in all ACS patients. Data are presented as mean ± SEM. *p*-values and *p < 0.001 *versus* non-ACS patients, Mann–Whitney test. **A–C.** While the increase in levels of miR-1 (**A**) and miR-499 (**C**) was relatively high in UA patients, miR-208a levels were only increased in NSTEMI patients (**B**).**D,E.** MiR-21 levels were comparable between NSTEMI and UA patients (**D**), the increase in levels of miR-146a (**E**) was most pronounced in NSTEMI patients.

Levels of both miR-1 and miR-499 were elevated in UA and NSTEMI patients when compared to non-ACS patients (*p* < 0.001; Fig 2A and C), while circulating miR-208a levels were significantly increased in NSTEMI patients only (Fig 2B). MiR-21 and miR-146a were significantly increased in both ACS subcategories (Fig 2D and E). For miR-208a and miR-146a, circulating miRNA levels seemed to be the highest in NSTEMI patients, while miR-21 levels were comparable between patients with unstable angina (UA) and NSTEMI patients. Levels of miR-1 and miR-499 were higher in UA patients.

### Circulating miRNA levels in suspected ACS patients with a negative hs-troponin at first presentation and in patients presenting within 3 h of symptom onset

In 194 suspected ACS patients (53.6% men, mean age 58.4 ± 12.7 years), initial hs-troponin levels were negative (<0.14 pg/ml). Of these, 23 (11.9%) were subsequently classified as having an ACS [median hs-troponin level 2.81 (IQR 1.0–6.1) pg/ml], including 18 UA and 5 NSTEMI patients. In this troponin-negative subgroup of suspected ACS patients, cardiac-related miRNAs were considerably higher in patients with ACS (Table 2). Circulating levels of miR-21 and miR-146a were also elevated.

**Table 2:** Relative expression of circulating miRNAs in serum of suspected ACS patients with a negative hs-troponin (*n* = 194) or with onset of symptoms <3 h (*n* = 152) compared to non-ACS patients (*n* = 226)

MicroRNA	Non-ACS	ACS patients with negative hs-troponin	*p*-value	ACS patients with symptom onset <3 h	*p*-value
miR-1	1.0 ± 0.2	14.9 ± 0.8	0.00	6.0 ± 0.6	0.00
miR-208a	1.0 ± 0.2	2.8 ± 0.7	0.03	2.4 ± 0.5	0.01
miR-499	1.0 ± 0.3	34.2 ± 0.7	0.00	24.0 ± 0.5	0.00
miR-21	1.0 ± 0.3	15.5 ± 0.8	0.00	11.0 ± 0.5	0.00
miR-146a	1.0 ± 0.4	6.2 ± 1.3	0.04	11.9 ± 0.8	0.00

Data are presented as mean ± SEM. *p*-value *versus* non-ACS patients.

In 152 suspected ACS patients (55.3% men, mean age 62.9 ± 14.5 years), the onset of chest pain was <3 h prior to presentation. In this subgroup, circulating levels of miR-1, miR-499 and miR-208a were also higher in ACS patients than non-ACS patients (Table 3). Mir-21 and miR-146a were markedly elevated in ACS patients as well.

**Table 3:** Diagnostic value of cardiac troponin and circulating microRNAs in suspected ACS patients

Marker	All patients (*n* = 332)		Hs-troponin negative patients (*n* = 194)		Patients with symptoms <3 h (*n* = 152)	
			
	AUC	95% CI	AUC	95% CI	AUC	95% CI
Cardiac troponin I	0.85	0.80–0.90	0.61	0.47–0.75	0.82	0.75–0.90
Cardiac hs-troponin T	0.86	0.82–0.91	0.74	0.62–0.85	0.86	0.80–0.92
miR-1	0.75	0.70–0.81	0.79^b^	0.69–0.89	0.67	0.28–0.77
miR-208a	0.61	0.54–0.67	0.64	0.52–0.77	0.62	0.53–0.72
miR-499	0.79	0.74–0.84	0.83^c^	0.76–0.90	0.79	0.71–0.86
miR-21	0.76	0.71–0.82	0.75	0.65–0.85	0.72	0.64–0.80
miR-146a	0.68	0.62–0.74	0.64	0.52–0.75	0.70	0.61–0.78
miR-1 + miR-499 + miR-21	0.89^a^	0.85–0.94	0.88^a^	0.83–0.94	0.82	0.76–0.89
Cardiac hs-troponin T with						
miR-1	0.90^a^	0.86–0.93	0.86^a^	0.79–0.92	0.88^c^	0.83–0.94
miR-208a	0.86	0.82–0.91	0.78	0.68–0.89	0.86	0.81–0.92
miR-499	0.90^a^	0.86–0.93	0.89^a^	0.83–0.94	0.90^a^	0.84–0.94
miR-21	0.89^a^	0.86–0.93	0.84^a^	0.77–0.91	0.89^b^	0.83–0.94
miR-146a	0.87	0.83–0.91	0.79	0.70–0.88	0.86	0.80–0.92

AUC, area under the ROC Curve; 95% CI, 95% confidence interval;

a *p* < 0.001,

b *p* = 0.03,

c *p* = 0.003 *versus* hs-troponin T.

### Additional diagnostic value of circulating microRNAs in ACS patients

The area under the ROC curve (AUC) of both cardiac troponin I [0.85; 95% confidence interval (CI) 0.80–0.90] and hs-troponin T (0.86; 95% CI 0.82–0.91) was higher than that of any of the miRs (Table 3). Of the miRs, miR-1, miR-499 and miR-21 had the highest AUC. When combining these miRs, the diagnostic value became significantly higher than that of hs-troponin T in the total suspected ACS patients and in the subgroup of suspected ACS patients with initially negative troponin levels (Table 3). Furthermore, combining miR-1, miR-499 or miR-21 with hs-troponin T significantly improved the diagnostic value by increasing the AUC to 0.90 (*p* < 0.001; Table 3).

As expected, the AUC of hs-troponin was low (0.61; 95% CI 0.47–0.75) in the subgroup of suspected ACS patients with initially negative hs-troponin levels (Table 3). Interestingly, the AUC of miR-1 and miR-499 were higher than any of the other miRs or cardiac (hs-)troponin. The AUC of miR-21 was comparable with hs-troponin (0.75; 95% CI 0.65–0.85). Addition of miR-1, miR-499 or miR-21 further increased the diagnostic discriminatory value significantly compared to hs-troponin alone (Table 3). Combining miR-1, miR-499 and miR-21 with hs-troponin T significantly improved the diagnostic value by increasing the AUC to 0.88 (95% CI 0.83–0.94). In patients with symptoms <3 h, a similar increase in AUC was observed with the addition of miR-1, miR-499 and miR-21 (Table 3).

### Circulating miRNAs are independent predictors for ACS

The clinical model with age, sex and cardiovascular risk factors resulted in an AUC of 0.72 (95% CI 0.66–0.78) for all suspected ACS patients (Table 4), which increased to 0.89 after addition hs-troponin T. Addition of miR-1, miR-499 or miR-21 to the clinical model with hs-troponin T significantly increased the AUC to 0.92 (*p* < 0.001). Importantly, after correction for the clinical model and (hs-)troponin, miR-1, miR-499 and miR-21 were still strong predictors for ACS as illustrated by their odds ratio (Table 4). Interestingly, the combination of the three miRs resulted in an even higher AUC of 0.94 (95% CI 0.92–0.97).

**Table 4:** AUCs and Odds ratios of miRNAs in suspected ACS patients in a clinical model (*n* = 332)

Marker	AUC	95% CI	OR^b^	95% CI
Clinical model (CM)	0.72	0.66–0.78	NA	NA
CM + cardiac troponin	0.88	0.85–0.92	NA	NA
CM + cardiac hs-troponin T	0.89	0.85–0.92	NA	NA
CM + cardiac hs-troponin T with				
miR-1	0.92^a^	0.90–0.95	1.30	1.17–1.42
miR-208a	0.89	0.85–0.93	1.16	1.03–1.30
miR-499	0.92^a^	0.89–0.95	1.28	1.18–1.40
miR-21	0.92^a^	0.89–0.95	1.28	1.18–1.39
miR-146a	0.90	0.87–0.94	1.14	1.08–1.21
miR-1 + miR-499 + miR-21	0.94^a^	0.92–0.97	NA	NA

CM, clinical model (age, sex, hypertension, hypercholesterolemia, family history, current and former smoking, diabetes mellitus, and history of myocardial infarction, PCI or coronary bypass surgery); AUC, area under the ROC curve; 95% CI, 95% confidence interval;

a *p* < 0.001 *versus* hs-troponin T. NA, not applicable;

b Adjusted for clinical model and cardiac hs-troponin T.

In patients with negative hs-troponin at presentation, addition of miR-1, miR-499 and miR-21 also resulted in a significant increase in AUC to 0.92 (Table 5), whereas miR-208a and miR-146a showed no additional effect. Despite negative hs-troponin levels at presentation, the combination of three miRs (miR-1, miR-499 and miR-21) resulted in a very high-AUC value of 0.96 (95% CI 0.93–0.99), again independent of other clinical risk factors (Table 5). In patients with symptom onset <3 h, these three miRs were the strongest predictors for ACS, increasing the AUC significantly as well (Supporting Information Table S3).

**Table 5:** AUCs and Odds ratios of miRNAs in suspected ACS patients with a negative hs-troponin in a clinical model (*n* = 194)

Marker	AUC	95% CI	OR^b^	95% CI
Clinical model (CM)	0.84	0.76–0.93	NA	NA
CM + cardiac troponin	0.85	0.77–0.94	NA	NA
CM + cardiac hs-troponin T	0.86	0.79–0.93	NA	NA
CM + cardiac hs-troponin T with				
miR-1	0.92^a^	0.87–0.96	1.44	1.19–1.73
miR-208a	0.87	0.78–0.95	1.12	0.95–1.35
miR-499	0.93^a^	0.87–0.99	1.38	1.19–1.61
miR-21	0.92^a^	0.88–0.97	1.34	1.15–1.55
miR-146a	0.86	0.78–0.93	1.06	0.97–1.15
miR-1 + miR-499 + miR-21	0.96^a^	0.93–0.99	NA	NA

CM, clinical model (age, sex, hypertension, hypercholesterolemia, family history, current and former smoking, diabetes mellitus, and history of myocardial infarction, PCI or coronary bypass surgery); AUC, area under the ROC curve; 95% CI, 95% confidence interval;

a *p* < 0.001 *versus* hs-troponin T; NA, not applicable;

b Adjusted for clinical model and cardiac hs-troponin T.

As a final step, we compared the combination of miR-1, miR-499 and miR-21 with the combination of three established markers of myocardial necrosis (hs-troponin, myoglobin and CK-MB) in the model with clinical risk factors (Supporting Information Table S4). In the total population, hs-troponin negative patients and early presenting patients, the combination of miRs performed better than the combination of these three necrosis markers. However, only in the hs-troponin negative population, this led to a statistically significant difference in AUC.

## DISCUSSION

Circulating miRNAs have been proposed as potentially useful novel biomarkers for detecting cardiac injury. We selected three miRNAs – miR-1, miR-499 and miR-208 – because of their known high expression in cardiac tissue to determine their diagnostic value in suspected ACS patients upon first presentation in the ED. Our results demonstrate that the levels of miR-1 and miR-499 are markedly elevated in patients with ACS compared to non-ACS patients (Fig 1A and C). These data confirm and extend previous reports showing high levels in patients with myocardial damage (Adachi et al, 2010; Ai et al, 2010; Cheng et al, 2010; Corsten et al, 2010; D'Alessandra et al, 2010; Kuwabara et al, 2011; Wang et al, 2010a, b). The increase in miR-1 levels was less pronounced than that of miR-499. This might explain why a previous study failed to demonstrate a significant increase in miR-1 levels compared to miR-499, which was markedly elevated in patients suffering from acute MI (Corsten et al, 2010). MiR-208a only increased slightly in the ACS group (Fig 1B). This is probably explained by the fact that miR-208a was undetectable in 86% of our samples (286/332), supported by an earlier study (Kuwabara et al, 2011). Also because of known difficulties in detecting miR-208a (and miR-208b) levels (Adachi et al, 2010; D'Alessandra et al, 2010; Widera et al, 2011), miR-208(a/b) appears to be the least suitable of the selected cardiac-expressed candidates.

In addition, we investigated the expression of miR-21 and miR-146a in ACS-suspected patients based on reports showing that these miRNAs were clearly related to cardiac injury or myocyte cell death and in patients suffering from ACS (Dong et al, 2009; Guo et al, 2010). Although further research is necessary to identify why these two miRNAs are upregulated, their involvement in cardiac disease has been established. Inhibition of miR-21 (depending on the anti-miR used) inhibited extensive fibrosis in the failing heart, mainly by decreasing fibroblast survival (Patrick et al, 2010; Thum et al, 2011). Additionally, miR-21 exerted cardioprotective effects as overexpression reduced myocardial cell death (Cheng et al, 2009; Dong et al, 2009) and miR-146a was reported to be upregulated in Doxorubicin-induced cardiotoxicity (Horie et al, 2010). Circulating levels of miR-21 (diastolic dysfunction) and miR-146a (diastolic dysfunction and viral myocarditis) showed no changes compared to control patients (Corsten et al, 2010). In line with our data, two recent studies reported increased miR-21 levels post-MI (Olivieri et al, 2012; Zile et al, 2011).

Analysis of miRNA levels in ACS subcategories revealed an increase of all miRNAs studied in UA and NSTEMI patients, except for miR-208a (only in NSTEMI patients). Interestingly, UA patients had relatively high levels of both miR-1 and miR-499, although this was not statistically different from NSTEMI patients. MiR-146a levels were the highest in NSTEMI patients; miR-21 levels were comparable between UA and NSTEMI patients. It is not unlikely that both miR-21 and miR-146a are subjected to more dynamic changes in the ischemic heart. This is supported by the fact that different profiling studies reported an increase in miR-21 and miR-146a after MI while miR-1 and miR-499 levels (already highly expressed) showed a more stable expression over time (Roy et al, 2009; van Rooij et al, 2008). Furthermore, levels of miR-146a in UA patients were below the mean level of all ACS patients, which might suggest that miR-146a could be helpful in discriminating NSTEMI from UA patients. Larger diagnostic studies will be necessary to validate these observations. These differences raise the question whether miRNAs are released on a regular base (*i.e.* intercellular communication upon stress signals) or simply reflect myocardial damage leading to uncontrolled leakage from damaged myocardium (Gupta et al, 2010).

It was previously reported that miRNAs in serum can be found in exosomes and microvesicular bodies (Wang et al, 2010a, b; Zampetaki et al, 2012). As their release can be affected by any form of cellular stress, cardiac necrosis is not necessary to evoke this response. This could explain why we observed elevated miRNA levels in patients found to have an ACS whose troponin was negative on initial presentation (*i.e.* patients with UA pectoris – in whom troponin remains negative by definition – and MI patients presenting too early after symptom onset for troponin to be positive). These observations suggest that miRNAs holds potential to improve the prognosis of both NSTEMI and UA patients who are known to benefit from swift initiation of treatment.

A recent study with a final diagnosis of ACS suggested that circulating miRs provide rather poor prognostic and diagnostic information (Widera et al, 2011). However, as this was designed as a prognostic study, a control group consisting of suspected ACS patients, but ultimately not having an ACS, was absent. A very recent study elegantly demonstrated that the transcoronary concentration of several miRs, including miR-499, are increased in patients undergoing catheterization (De Rosa et al, 2011). Importantly, our data further extend these reports, as we included non-ACS patients and analysed blood collected at first presentation. The increase in AUC when adding miRNAs to the current ACS biomarker high-sensitive troponin, the fact that a combination of miRNAs resulted in a higher AUC than high-sensitive troponin, and the AUC of single miRs in the troponin-negative population, underscore the potential diagnostic value of these novel biomarkers in clinical practice. Finally, in a clinical model with important co-variates including age, sex and cardiovascular risk factors, miRNAs were shown to be strong independent predictors of ACS. Importantly, circulating miRs were not significantly correlated with age, hypertension and cholesterol levels.

Especially three of the investigated miRs (miR-1, miR-499 and miR-21) seem very promising, as their combined diagnostic performance is statistically better than hs-troponin. Furthermore, this miR-combination also led to higher AUC values than the combination of three myocardial necrosis markers (hs-troponin, myoglobin and CK-MB).

The main strength of our study is the evaluation of miRNAs isolated from serum collected immediately upon presentation to the ED in a large group (*n* = 332) of patients, in a clinically relevant setting: *i.e.* patients suspected of an ACS in whom clinical and ECG findings are inconclusive. Apart from comparing miRNA levels in patients with and without an ACS, we also evaluated the added diagnostic value of miRNA compared to high-sensitive troponin and in a clinical model with relevant co-variates. Importantly, a combination of three miRNAs showed better diagnostic performance than hs-troponin T alone.

Several limitations have to be taken into account. From the original 470 patients, 138 were excluded because of poor RNA integrity indicating that the starting material was not optimal for our analysis; but importantly, the patient characteristic of these 138 patients and the 332 included patients were similar, indicating that this is very unlikely to have changed our findings. The material was collected in 2007 when optimal methods for processing and storage of serum for miRNAs isolation was largely unknown. Measurement of circulating miRNAs requires RT-PCR, which is currently the limiting factor in terms of rapid detection. However, as miRNAs have clear diagnostic potential, newer and less expensive techniques to detect serum miRNA levels more rapidly can be expected in the near future. This would also facilitate additional research investigating the incremental value of circulating miRs when added to a clinical risk score of ACS like the GRACE Risk Score or HEART score, which might be clinically very relevant (Backus et al, 2011).

Given the inherent limitations of our study, they remain to be confirmed in a larger, prospective, multicentre diagnostic study and in a large independent population of hs-troponin negative patients.

In patients suspected of ACS presenting to the ED within 24 h of symptom onset, circulating microRNA levels (miR-1, miR-208a, miR-499, miR-21 and miR-146a) are higher in those with an ACS and are already increased in suspected ACS patients with initially negative troponin levels and in those presenting within 3 h of symptom onset. Addition of miR-1, miR-499 or miR-21 significantly increased the diagnostic value compared to hs-troponin T and these three miRs were independent predictors of ACS. Interestingly, the combination of these three miRs resulted in a higher AUC than hs-troponin T, including the hs-troponin negative patients. These findings demonstrate that circulating miRNAs hold great potential as novel early biomarkers of cardiac injury. MiRNAs might be useful for better management of suspected ACS patients, in particular those with UA pectoris and NSTEMI in whom diagnostic uncertainty is high.

## MATERIALS AND METHODS

### Study population

The present study a single-centre, prospective diagnostic study among patients presenting to the ED within 24 h of onset of chest pain suggestive of ACS. Patients were enrolled at the ED of the Meander Medical Center (Amersfoort, the Netherlands) between May 2007 and November 2007 ST-elevation myocardial infarction (STEMI) – these patients underwent immediate PCI – or refusal to provide informed consent were the only exclusion criteria, 470 patients were evaluated. All included patients provided written informed consent, the protocol was approved by the local ethics committee and the study was conducted in accordance with the Declaration of Helsinki. RNA extraction and analysis of stored serum samples was performed in all 470 patients. Despite several attempts to isolate good quality RNA, we had to exclude 138 patients because of poor RNA integrity, mostly due to errors in sample processing at time of collection and/or storage. The current analyses thus included 332 patients with chest pain. Importantly, the mean age (61.7 ± 14.9) and median onset of symptoms (3.2 h; IQR 2.0–6.7) in the 138 excluded patients and in the 332 included patients were similar (Supporting Information Table S5).

### Routine clinical assessment

All patients underwent routine clinical assessment, including medical history, physical examination, serial 12 lead ECGs, pulse oxymetry and chest radiography. Immediately upon presentation, venous blood was drawn to determine cardiac Troponin I (cTnI) and other routine laboratory parameters. At the same time, additional samples were taken and stored for future analyses, including serum miRNA analysis (Supporting Information Fig S1). cTnI levels were measured by sandwich chemoluminescence immunoassay (Synchron Lxi 725 integrated clinical chemistry, Beckman Coulter). The lower detection limit for cTnI was 0.01 ug/L and the cut-off level for positivity was ≥0.04 µg/L. High-sensitive cardiac Troponin T (hs-cTnT) was determined *post hoc* in serum stored at −80°C. The lower detecting limit for hs-cTnT was 3 pg/ml, measured by the Elecsys troponin T high-sensitive assay fourth generation (Roche Diagnostics) with the 99th-percentage cut-off point of ≥14 pg/ml.

### Blood processing

Blood samples were collected via a venous puncture at the time of presentation at the ED and 5 ml of blood was collected in a standard serum tube (BD #368968). Blood was centrifuged for 10 min after which serum was aspirated, aliquoted in RNAse-free tubes and stored at −80°C until further processing.

### RNA isolation

RNA was extracted of fixed volumes of serum (250 µl) using TRIzol LS reagent (Invitrogen 10296-028) as described previously (Chen et al, 2008; Kuwabara et al, 2011). Genomic DNA contamination was eliminated using DNAse I kit (Ambion) and RNA concentration quantified with a spectrophotometer (NanoDrop ND-1000, Thermo Scientific, Wilmington, DE). The RNA integrity of small RNAs was determined using small RNA Chip analysis (5067-1548) for an Agilent Bioanalyzer 2100 (Agilent Technologies).

### Quantitative RT-PCR

A total of 20 ng DNA-free RNA was used as input in the reverse transcription reaction (RT) using the TaqMan miRNA Reverse Transcription Kit and miRNA-specific primers (Applied Biosystems) as described previously (Kroh et al, 2010). Real-time PCR (RT-PCR) amplification was performed in duplicate using 1:5 diluted RT products for the following miRNAs: miR-1 (#002222), miR-208a (#000511), miR-499 (#002427), miR-21 (#000397) and miR-146a (#000468). Currently, no consensus exists on the use of an internal control for RT-PCR analysis of circulating miRNAs. All RT-PCR data were standardized to U6, which was used by others before (Tijsen et al, 2010; Zile et al, 2011), moreover, U6 levels did not differ between ACS and non-ACS patients. After normalization for RNU6, relative gene expression was calculated by the ΔΔ*C*_t_ method (Noort et al, 2012; Oerlemans et al, 2010).

**The paper explained**

PROBLEM:

Currently, the diagnosis of ACS is based on elevation of (high-sensitive) cardiac troponins, which are not consistently elevated within the first hours after symptom onset. In daily clinical practice, typical symptoms together with ST segment elevation on the ECG is only present in a minority of patients presenting to the emergency department (ED), making the early diagnosis of ACS a challenge. New diagnostic markers to improve early recognition of ACS are therefore required, for which microRNAs were previously investigated. MicroRNAs are short, non-coding small RNAs that regulate the protein expression by translational repression or degradation of messenger RNAs. Previous studies investigating the role of circulating microRNAs in ACS were based on small patient numbers, performed no comparison with established markers of cardiac injury or did not have appropriate controls.

RESULTS:

We determined the potential diagnostic value of circulating microRNAs as novel early biomarkers in 332 suspected ACS patients on presentation to the ED. Circulating microRNA levels (miR-1, miR-208a, miR-499, miR-21 and miR-146a) were higher in patients diagnosed with an ACS. Moreover, these miRNAs were also increased in patients diagnosed with ACS when initial troponin was still negative or with symptom onset <3 h. Especially miR-1, miR-499 and miR-21 significantly increased the diagnostic value in all suspected ACS patients when added to high-sensitive troponin T (AUC 0.90). MiR-1, miR-499 and miR-21 were strong predictors of ACS independent of clinical co-variates, including patient history (age, sex, previous MI, PCI or surgery) and cardiovascular risk factors. Interestingly, the combination of these three miRs resulted in a significantly higher AUC of 0.94 than hs-troponin T (0.89).

IMPACT:

Using a real-world population of suspected ACS patients presenting to the ED, our study shows that circulating miRNAs hold great potential as novel early biomarkers of cardiac injury. These findings have important clinical implications as miRNAs might be useful for better management of suspected ACS patients than can be achieved by the current biomarker of choice, high-sensitive troponin T. This is of particular interest in patients with unstable angina pectoris and NSTEMI in whom diagnostic uncertainty is high.

### Outcome

The primary outcome of this study was ACS, including UA and non-ST-elevation myocardial infarction (NSTEMI). Final diagnosis was made by an expert panel of three cardiologists, based on all available clinical information including serial troponin measurements, serial ECG findings, coronary angiography, echocardiography, cardiac exercise tests and information from hospital discharge letters.

Early invasive coronary angiography was performed in 30 (36.6%) out of 82 NSTE-ACS patients. Most important reasons were recurrent or non-resolving symptoms (*n* = 10), dynamic ECG changes (*n* = 8), prior CABG or recent PCI (*n* = 11) and ventricular tachycardia (n = 1). From the remaining 52 patients, in 38 (73.1%) patients symptoms resolved upon medicinal treatment. Of the 14 patients with non-resolving symptoms, 4 patients requested conservative therapy, in 5 patients a conservative strategy was chosen considering co-morbidity (aged >85 years, renal failure, CVA) and in 5 patients no coronary angiography was performed due to known multivessel disease (>2 coronary arteries) or graft or stent failure requiring CABG surgery.

The presence of ACS was determined according to the universal definition of myocardial infarction (Thygesen et al, 2007). NSTEMI was diagnosed when there was evidence of myocardial necrosis together with clinical signs and symptoms of myocardial ischemia, according to the current guidelines (Anderson et al, 2011; Hamm et al, 2011). Myocardial necrosis was diagnosed by a rising and/or fall in cardiac troponin with at least one value above the 99th percentile. UA was diagnosed in the presence of clinical signs and symptoms of myocardial ischemia, including cardiac exercise test, without elevation of cardiac biomarkers.

### Statistical analysis

Data are presented as mean ± SEM unless otherwise indicated. Differences between groups were analysed by Mann–Whitney U Test or Kruskal–Wallis Test (>2 groups) when appropriate using Bonferroni correction for multiple comparisons. Spearman rank correlation was used to compare patient characteristics with circulating microRNAs. Receiver-operating-characteristic (ROC) curves were constructed to of each miRNA using the area under the ROC curve (AUC) to assess their ability to diagnose ACS (Reichlin et al, 2009). To compare diagnostic discriminatory ability of miRNAs with cardiac (hs-)troponin, likelihood ratio tests were performed after logistic regression to assess the additional diagnostic value of miRNA to the information provided by (hs-) troponin. Multivariate logistic regression was used to investigate whether miRNAs were independent predictors of ACS after adjustment for relevant co-variates including patient history (age, sex, previous MI, PCI or surgery) and cardiovascular risk factors (hypertension, hypercholesterolemia, family history, current and former smoking and diabetes mellitus). All tests were two-sided, using a significance level of *p* < 0.05 (SPSS Statistics v17, Chicago, United States).

## Supplementary material

Detailed facts of importance to specialist readers are published as ”Supporting Information”. Such documents are peer-reviewed, but not copy-edited or typeset. They are made available as submitted by the authors.
