# Supplementary material for: Early assessment of acute coronary syndromes in the emergency department: the potential diagnostic value of circulating microRNAs
Source: EMBO Mol Med. 2012 Oct 1;4(11):1176–85. doi: 10.1002/emmm.201201749 (PMC3494874; doi:10.1002/emmm.201201749)
Supplement: Supplementary file 1 [file emmm0004-1176-SD1.pdf]

EMBO
Molecular Medicine

Manuscript EMM-2012-01749

Early assessment of acute coronary syndromes in the emergency department: the potential diagnostic value of circulating microRNAs

Martinus I.F.J. Oerlemans, Arend Mosterd, Marieke S. Dekker, Evelyn A. de Vrey, Alain van Mil, Gerard Pasterkamp, Pieter A. Doevendans, Arno W. Hoes, Joost P.G. Sluijter

Corresponding author: Joost Sluijter, University Medical Center Utrecht

Review timeline:

Submission date:	24 March 2012
Editorial Decision:	02 May 2012
Resubmission:	16 July 2012
Editorial Decision:	16 August 2012
Revision received:	23 August 2012
Accepted:	27 August 2012

Transaction Report:

(Note: With the exception of the correction of typographical or spelling errors that could be a source of ambiguity, letters and reports are not edited. The original formatting of letters and referee reports may not be reflected in this compilation.)

1st Editorial Decision 02 May 2012

Thank you for the submission of your manuscript to our editorial offices. We have now received feedback from 2 out of 3 reviewers whom we asked to evaluate your manuscript. Given that both reviewers provide very similar recommendations, we prefer to make a decision now in order to avoid further delay in the process.

As you will see from the enclosed reports, neither referee supports publication of the manuscript in EMBO Molecular Medicine in this current state. Although they find the topic of the study potentially intersting, they also feel that a considerable amount of additional analyses and extra experiments shoud be done for the paper to be conclusive.

I would prefer not to repeat their individual points of criticism in this letter, but clearly both of them point to a number of serious analytical and technical shortcomings of your study, and feel that given these limitations, your results would appear to be of rather limited general significance.

Given these negative opinions and the fact that the EMBO Molecular Medicine can only afford to accept papers which receive enthusiastic support from a majority of referees, I am afraid I see little choice but to return the manuscript to you at this point with the decision that we cannot offer to publish it.

In light of the potential interest of the findings though, we would have no objection to consider a new manuscript on the same topic if at some time in the near future you obtained data/analysis that would considerably strengthen the message of the study and address the referees concerns in full. To be completely clear, however, I would like to stress that if you were to send a new manuscript this

would be treated as a new submission rather than a revision and would be reviewed afresh, in particular with respect to the literature and the novelty of your findings at the time of resubmission. If you decide to follow this route, please make sure you nevertheless upload a letter of response to the referees' comments.

At this stage of analysis, however, I am sorry to have to disappoint you. I nevertheless hope, that the referee comments will be helpful in your continued work in this area (I will forward the latest report to you if we ever get it) and I thank you for considering EMBO Molecular Medicine.

Yours sincerely,

Editor
EMBO Molecular Medicine

***** Reviewer's comments *****

Referee #2:

In this study by Oerlemans, Mosterd et al., the authors analyzed the role of the circulating microRNAs miR1, 21, 146a, 208a and 499 in a rather large study cohort of 332 acute coronary patients (ACS) as diagnostic biomarkers directly in the chest pain unit. They found, that levels of all investigated MiRs were increased in case of the following diagnosis of NSTE-ACS and MiR levels were independent to other clinical co-variates and superior to the gold-standard hsTroponin T. Furthermore the authors evaluated for the combination of a "multi-miR-Panel" with miR1, 21 and 499 a better diagnostic ability than for hsTnT alone.
The approach of evaluating the role of circulating miRs in a large cohort of chest-pain-unit patients is interesting, especially considering the aspect of the early NSTE-ACS-diagnosis by miRs, and confirms other recently published studies (especially the work of Widera et. Al; J Mol Cell Cardiol. 2011 Nov;51(5):872-5 based on 444 ACS patients). The paper is well written and uses adequate statistical methods.

Some points merit discussion:

1. I have a lot of problems with your study collective: You collected plasma samples directly in the chest pain unit: Are there no patients with a STE-ACS? Why didn¥t you investigate them as an additional study group (take blood from arterial sheath in cat-lab before PCI for example)? For my opinion, if miR1, 21 and 499 are really high sensitive myonecrosis-biomarkers I would like to see the levels of STEMI-patients, which should have highest levels.
2. You use ACS-definition as "gold diagnostic standard" for your study: It seems that it¥s based on "traditional Troponin measurements": If this is really the case - with respect to actual studies for high-sensitive Troponin assays in early ACS diagnosis (see for example: Keller et al. NEJM 2009) - your study has an important limitation: Than I would suggest a completely new analysis, based on the post-hoc measured hsTnT levels: You analyzed the subgroup of patients with negative Troponin at admission to the chest pain unit, and a later troponin-positive-based ACS diagnosis: That¥s the most interesting part of your study, but it¥s also significantly limited if you use the traditional Troponin measurement: What¥s about clinical characteristics of patients with/without positive TnT at admission and later ACS diagnosis? Are there differences?
3. Did all your patients get the same standard therapy by emergency doctors? Can you exclude that emergency doctors didn¥t treat patients with a higher possibility for an ACS with Heparin and/or dual antithrombotic therapy: Did you check, that this therapy didn¥t influence your miR-levels, and causes the selective measurable upregulation in case of ACS?
4. Data analysis: I really don¥t like your statistical approach: Table 3: On the one hand calculating AUC evaluating the diagnostic performance of a new biomarker is antique, please calculate C-statistics (vgl. Zethelius et al.; NEJM 2008): On the other hand: How did you determine the AUC for the miRs? Did you use a specific cut-off-point? You have to explain that, especially because this is the basis for your key-message. Table 4: You compare AUC of a clinical model based on simple cardiovascular risk factors with biomarkers and use this for the situation of an acute coronary syndrome patient? If you really want to compare MiRs with a score, please use an established ACS-score!

5. If you calculate the diagnostic performance of a "multi-miR-panel" reflecting myonecrosis by three different markers please compare it with three established myocardial necrosis factors, for example hsTnT+ Myoglobine+CK-MB.

Referee #3 (Comments on Novelty/Model System):

The ms studies circulating microRNAs as novel biomarkers in acute coronary syndrome (ACS). Authors conclude that the combination of three microRNAs result in a significantly higher AUC than the current gold standard biomarker hs-troponin T.
The ms is clinically relevant. Obviously analyses have been well performed.
There are, however significant limitations. My major point is that the validity of the data critically depends on the definition of ACS which needs invasive or non invasive coronary angiography. Alternatively outcome analysis may be presented. If the authors would be able to provide one or the other, the ms would be interesting and relevant enough for publication.

Referee #3 (Other Remarks):

The ms studies circulating microRNAs as novel biomarkers in acute coronary syndrome (ACS). Authors conclude that the combination of three microRNAs result in a significantly higher AUC than the current gold standard biomarker hs-troponin T.
I have the following comments:
1) The validity of the data critically depends on the definition of ACS which needs invasive or non invasive coronary angiography. Alternatively, outcome analysis may be presented.
2) The authors had to exclude 138 patients because of poor RNA quality, this may cause systematic error.
3) According to table 1, age, hypertension , and cholesterol levels were different between groups and could be confounders. Please discuss and provide calculations.

Resubmission 16 July 2012

We appreciate the helpful and constructive comments from the reviewers. We have attempted to carefully address point-to-point the issues raised by reviewers #2 and #3 (listed in Italic) with additional text and experiments and believe that the manuscript has been considerably strengthened as a result. Applied changes to the manuscript were underlined.

Referee #2:

In this study by Oerlemans, Mosterd et al., the authors analysed the role of the circulating microRNAs miR1, 21, 146a, 208a and 499 in a rather large study cohort of 332 acute coronary patients (ACS) as diagnostic biomarkers directly in the chest pain unit. They found, that levels of all investigated MiRs were increased in case of the following diagnosis of NSTE-ACS and MiR levels were independent to other clinical co-variates and superior to the gold-standard hsTroponin T. Furthermore the authors evaluated for the combination of a "multi-miR-Panel" with miR1, 21 and 499 a better diagnostic ability than for hsTnT alone.
The approach of evaluating the role of circulating miRs in a large cohort of chest-pain-unit patients is interesting, especially considering the aspect of the early NSTE-ACS-diagnosis by miRs, and confirms other recently published studies (especially the work of Widera et. Al; J Mol Cell Cardiol. 2011 Nov;51(5):872-5 based on 444 ACS patients). The paper is well written and uses adequate statistical methods.

We appreciate the reviewer’s description of our study as interesting and well-written.

Some points merit discussion:
1. I have a lot of problems with your study collective: You collected plasma samples directly in the chest pain unit: Are there no patients with a STE-ACS? Why didn't you investigate them as an

additional study group (take blood from arterial sheath in cat-lab before PCI for example)? For my opinion, if miR1, 21 and 499 are really high sensitive myonecrosis-biomarkers I would like to see the levels of STEMI-patients, which should have highest levels.

To investigate the potential diagnostic value of circulating miRs, we wanted to focus on the group ACS patients in which diagnostic uncertainty was the highest (i.e NSTEMI and UA patients). In patients with ST-elevation AMI, clinical assessment and ECG provide enough information for the diagnosis of STEMI followed by revascularization, which we therefore not used in our study population. As mentioned by the reviewer, early NST-ACS diagnosis using circulating miRS is therefore more interesting than its use in STEMI patients. However, it can be expected that circulating miR levels are very high in STEMI patients, which was investigated and observed by many others before, thereby using blood from patients just prior to primary PCI. These miRNAs showed increases up to 1600-fold compared to control patients (Wang et al, EHJ 2010, PMID 20159880; Cheng et al, Clinical Science 2010, PMID 20218970; Corsten et al, Circ Genetics 2010, PMID 20921333)

2a. You use ACS-definition as "gold diagnostic standard" for your study: It seems that it's based on "traditional Troponin measurements": If this is really the case - with respect to actual studies for high-sensitive Troponin assays in early ACS diagnosis (see for example: Keller et al. NEJM 2009) - your study has an important limitation: Than I would suggest a completely new analysis, based on the post-hoc measured hsTnT levels: You analysed the subgroup of patients with negative Troponin at admission to the chest pain unit, and a later troponin-positive-based ACS diagnosis: That's the most interesting part of your study, but it's also significantly limited if you use the traditional Troponin measurement: What's about clinical characteristics of patients with/without positive TnT at admission and later ACS diagnosis? Are there differences?

The reviewer is right that high-sensitive troponin, especially levels at presentation and their absolute/relative changes within the first hour, greatly reduce time to diagnosis and thus intervention, which is stated in the latest ESC guidelines (2011), and is well-illustrated by the study of Keller et al (NEJM 2009) and many others. However, in those studies as well as in ours, final diagnosis of ACS was adjudicated by an expert panel based on al clinically available data including patient history, physical examination, results of laboratory testing including cTnI values, radiological testing, serial ECGs, echocardiography, cardiac exercise test and coronary angiography. To our opinion final diagnosis will therefore not differ, as adjudicated by the expert panel. This was illustrated recently by Haaf et al (Circulation 2012, PMID 22623715), showing a similar amount of patients diagnosed as AMI with high-sensitive vs. conventional troponin, although it can help discriminating AMI from acute cardiac non-coronary artery disease in an earlier stage, next to earlier diagnosis of ACS patients.

For all other analyses, we already compared the diagnostic information provided by circulating miRs with hs-troponin instead of conventional troponin. With respect to the evaluation of troponin-negative patients, the reviewer is right that it is better to use hs-troponin instead of the conventional troponin for classification.
As requested by the reviewer, we redefined troponin-negative patients as patients with hs-troponin <0.14pg/ml and performed a new analysis of this subgroup of patients, leading to n=194 patients compared to 209 patients when using conventional troponin. Also in this subgroup of hs-troponin negative patients, all circulating miRs were significantly higher in patients with ACS compared to non-ACS patients (Table 2). Furthermore, miR-1, -21 and -499 significantly added diagnostic performance on top of hs-troponin alone and the combination of these three miRs resulted in an AUC of 0.88 (95% CI 0.83-0.94). In a clinical model with relevant co-variates, these three miRs both individually as combined , significantly increased AUC up to 0.96 (95% CI 0,93-0,99) on top of hs-troponin.
<u>We replaced the old analysis using conventional troponin with the above one, using hs-troponin for the identification of hs-troponin negative patients.</u>

Considering the clinical characteristics between hs-troponin negative and positive patients who will develop ACS, we found significant differences in troponin and hs-troponin levels as expected. Also, current smoking and parental cardiovascular disease or history of cardiovascular disease was higher in the hs-troponin positive subgroup. Due to the extended nature of tables in the manuscript we decided not to include this table in the manuscript but to provide it to the reviewer.

	trop pos	trop-	P
N (%)	83	23	
Age (yrs)	69,41 13,1	66,22 10,4	0.22
Male sex (% male)	58 (69,9)	12 (52.2)	0.14
Onset of chest pain, median (IQR)	3.5 (1.8 - 8.7)	2.5 (1.3 - 5.3)	0.95
Risk factors			
Current smoker	23 (27.7%)	1 (4.3%)	0.02
Former smoker	24 (28.9%)	8 (34.8%)	0.61
Hypertension	45 (54.2%)	16 (69.6%)	0.33
Hypercholesterolemia	32 (38.6%)	12 (52.5%)	0.34
Diabetes Mellitus	18 (21.7%)	3 (13.0%)	0.56
Body mass index (kg/m²)	25.92 0.7	26.09 1.1	0.57
Parental CVD	29 (34.9%)	15 (65.2%)	0.01
History of CVD	44 (52.4%)	18 (78.3%)	0.03
Cardiac Troponin I (μg/L)	0.11 (0.06 - 0.26)	0.02 (0.01 - 0.03)	0.00
Cardiac high-sensitive Troponin T (pg/mL)	41.2 (18.7 - 108.2)	7.25 (3.1 - 11.3)	0.00

3. Did all your patients get the same standard therapy by emergency doctors? Can you exclude that emergency doctors didn't treat patients with a higher possibility for an ACS with Heparin and/or dual antithrombotic therapy: Did you check, that this therapy didn't influence your miR-levels, and causes the selective measurable upregulation in case of ACS?

As blood collection was performed immediately upon presentation to the emergency department, medical treatment in terms of heparin or dual antiplatelet therapy that started after presentation, could not have influenced our data on circulating miRs. Furthermore, we looked whether miR-levels in ACS patients were correlated with aspirin, clopidogrel or heparin treatment (see table below, Spearman's correlation). No significant correlations were found, we added these results to our manuscript (page 5, lines 17-20; supporting information Table S2.

Therapy	miR-1	P	miR-208a	P	miR-499	P	miR-21	P	miR-146a	P
**Aspirin**	0.16	0.11	-0.03	0.79	0.08	0.41	-0.18	0.08	-0.10	0.06
**Clopidogrel**	0.11	0.27	-0.19	0.06	-0.06	0.57	-0,14	0.17	-0.11	0.26
**Heparin**	0.04	0.69	0.09	0.39	-0.10	0.34	-0.05	0.60	0.03	0.78

Moreover, to make our work-flow of patients from moment of presentation to the emergency department until quantification of miRs more clear, we added an overview illustrating our study setup as a supporting figure (see supporting information Fig. S1).

4a. Data analysis: I really don't like your statistical approach: Table 3: On the one hand calculating AUC evaluating the diagnostic performance of a new biomarker is antique, please calculate C-statistics (vgl. Zethelius et al.; NEJM 2008): On the other hand: How did you determine the AUC for the miRs? Did you use a specific cut-off-point? You have to explain that, especially because this is the basis for your key-message.

For determination of AUCs of the miRs, we assessed area under the receiver-operating-characteristic curve (AUC). Furthermore, as no established cut-off exists with respect to circulating miRs, AUC seems to be the best choice to evaluate the diagnostic performance. After careful reading of the paper and discussion with our statisticians, we feel that the method described in the paper by Zethelius et al is more appropriate when assessing prognostic modelling to predict future outcomes (Cox regression / hazard model). This makes sense as both this particular paper (prediction of death from cardiovascular causes) as well as the paper which is referred to in the methods (Pencina et al, Stat Med. 2008, PMID 17569110) calculate risks on future events over time. As we evaluate the diagnostic value of circulating miRs, we are interested in cross-sectional

analyses without future events over time. Therefore, we and many others having a similar research question, use ROC curve analysis to evaluate the diagnostic value (Keller et al, NEJM 2009, PMID 19710485; Reichlin et al, NEJM 2009, PMID 19710484; Corsten et al, Circ Genetics 2010, PMID 20921333; Wang et al, EHJ 2010, PMID 20159880; Olivieri et al, Int J Cardio 2012, PMID 22330002; Haaf et al, Circulation 2012, PMID 22623715).
We described our analyses more clearly in the methods section (page 14, lines 16-20).

4b. Table 4: You compare AUC of a clinical model based on simple cardiovascular risk factors with biomarkers and use this for the situation of an acute coronary syndrome patient? If you really want to compare MiRs with a score, please use an established ACS-score!

Whether a new biomarker could add diagnostic information to an established ACS-score is a different question than what we are aiming for in the current study. Beside the fact that again these scores (i.e. Grace Risk Score) generally predict both MI and related mortality (prognostic, future event), a risk factor should be evaluated and validated to begin with. However, we not only assessed the individual diagnostic value of circulating miRs (univariate), but also looked at their additional value in a model consisting of traditional risk factors (multivariate). We feel that this is an important step to select novel biomarkers that can truly provide diagnostic information that is clinically relevant. After validation in another large independent cohort, it would be very interesting to investigate the incremental value in an established ACS risk score system such as the HEART score (Backus et al, Curr Cardiol Rev 2011, PMID 22294968).
We added a summary of this part into our discussion section (page 10, lines 18-20).

5. If you calculate the diagnostic performance of a "multi-miR-panel" reflecting myonecrosis by three different markers please compare it with three established myocardial necrosis factors, for example hsTnT+ Myoglobine+CK-MB.

We thank the reviewer for this interesting idea. We compared the diagnostic performance of the most promising three miR-1, -21 and -499 directly with hsTnT+ Myoglobine+CK-MB in the total population, hs-troponin negative population, and in early presenting patients (<3hrs) using the clinical model as described earlier to correct for relevant co-variates (see table below). Both in the total population as in the different subgroups, AUC of the three miRs is higher than that of the myocardial necrosis markers. However, it did not always reach statistical significance (early presenters), although a strong tendency was observed in the total population.
We added these results to our manuscript (page 7, lines 13-18; supporting information Table S4) and discussion section (page 10, lines 1-4).

Supporting information Table S4 – Diagnostic value the combined miRs and myocardial necrosis markers in suspected ACS patients

	All patients (n=332)		Hs-troponin negative patients (n=194)		Patients with symptoms <3 hours (n=152)	
Marker	AUC	95% CI	AUC	95% CI	AUC	95% CI
Myoglobin/CK-MB/hs-trop	0.87	0.83-0.92	0.87	0.81-0.94	0.89	0.84-0.95
miR-1/ miR-499/miR-21	0.89	0.86-0.93	0.94*	0.90-0.99	0.92	0.88-0.97
P	0.41		0.001			0.07

Referee #3 (Comments on Novelty/Model System):

The ms studies circulating microRNAs as novel biomarkers in acute coronary syndrome (ACS). Authors conclude that the combination of three microRNAs result in a significantly higher AUC than the current gold standard biomarker hs-troponin T. The ms is clinically relevant. Obviously analyses have been well performed. There are, however significant limitations. My major point is that the validity of the data critically depends on the definition of ACS which needs invasive or non invasive coronary angiography. Alternatively outcome analysis may be presented. If the authors would be able to provide one or the other, the ms would be interesting and relevant enough for publication.

The ms studies circulating microRNAs as novel biomarkers in acute coronary syndrome (ACS). Authors conclude that the combination of three microRNAs result in a significantly higher AUC than the current gold standard biomarker hs-troponin T.
I have the following comments:
1) The validity of the data critically depends on the definition of ACS which needs invasive or non invasive coronary angiography. Alternatively, outcome analysis may be presented.

As pointed by the reviewer, the question of whether to perform early coronary angiography is an important management decision in the ED. In case of STE-ACS, we considered all patients a candidate for invasive coronary angiography. For NSTE-ACS, invasive strategy is recommended in high-risk patients (troponin rise or fall, dynamic ST- or T-wave changes) and patients with recurrent symptoms or hemodynamic instability (ESC Guidelines 2011).
In our study population, 30 (36.6%) out of 82 NSTE-ACS patients received early invasive coronary angiography. Most important reasons were recurrent or non-resolving symptoms (n=10), dynamic ECG changes (n=8), prior CABG or recent PCI (n=11) and ventricular tachycardia (n=1).
From the 52 patients who did not receive early invasive coronary angiography, in 38 patients (73.1%) symptoms resolved upon medicinal treatment. Of the 14 patients with non-resolving symptoms, 4 patients requested conservative therapy, in 5 patients a conservative strategy was chosen considering co-morbidity (aged >85 years, renal failure, CVA) and in 5 patients no coronary angiography was performed due to known multivessel disease (>2 coronary arteries) or graft or stent failure requiring CABG surgery.
<u>We added this to our methods section (page 13, lines 28-30; page 14, lines 1-10, outcome paragraph).</u>

2) The authors had to exclude 138 patients because of poor RNA quality, this may cause systematic error.

We performed several RNA isolations of the 138 excluded patients, but we were not able to perform a good RT-PCR on these samples. We decided to look into detail what the reason for this was using a using small RNA Chip analysis (5067-1548) for an Agilent Bioanalyzer 2100. As illustration, we provided the results of the analysis in below. As depicted there, the miRNA region (between the blue lines) showed fluorescent signal in samples that gave no problems (A), while in excluded samples almost no fluorescence was present (flat line) (B). Although we could not find a reason for this difference, at least we were able to demonstrate that those 138 samples contained poor (mi)RNA content in a very low concentration. We can only speculate on possible explanations. One might be that that miRNAs stay bound to serum proteins, which are present in very high concentrations, and cannot be isolated, leading to some kind of scavenging effect. <u>We added a summary of the above as well to our manuscript (page 12, lines 11-15))</u>

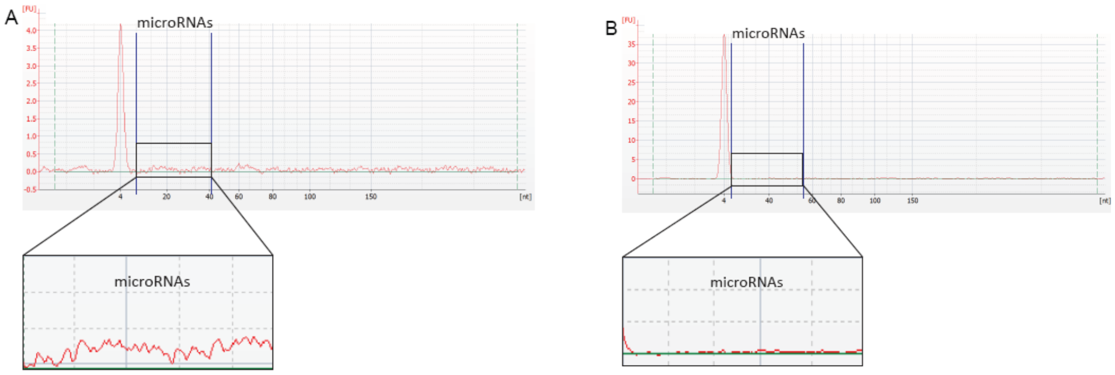

Importantly, we carefully compared the baseline characteristics of the excluded patients (n=138) vs. the included patients (n=332) suspected of ACS. As depicted in the table below, no differences were found.
<u>We added the table to the Supporting Information Table S5 .</u>

Characteristic	Included	Excluded	P*
N (%)	332 (70.6)	138 (29.4.6)	
Age (yrs)	62.9±14.4	61.7±14.9	0.42
Male sex (% male)	190 (57.2)	73 (53.5)	0.44
Onset of chest pain, median (IQR)	3.2 (1.8-8.0)	3.2 (2.0-6.7)	0.89
Risk factors			
Current smoker	86 (26.1)	39 (28.2%)	0.45
Former smoker	103 (31.5)	34 (24.6%)	0.11
Hypertension	151 (45.8)	57 (41.3)	0.25
Hypercholesterolemia	109 (33.0)	42 (30.4%)	0.42
Diabetes Mellitus	52 (15.8)	22 (15.9%)	0.90
Body mass index (kg/m²)	26.3±5.8	26.7±4.8	0.69
Parental CVD	133 (40.7)	50 (36.2%)	0.24
History of CVD	159 (47.9)	67 (48.6%)	0.91
Cardiac Troponin I (μg/L)	0.03 (0.02-0.05)	0.03 (0.02-0.04)	0.83
Cardiac hs-Troponin T (pg/mL)	6.0 (2.2-18.7)	5.3 (2.8-18.2)	0.77

3) According to table 1, age, hypertension , and cholesterol levels were different between groups and could be confounders. Please discuss and provide calculations.

As in many studies, investigating new biomarkers in ACS patients versus control patients, several characteristics are different between groups and could be potential confounders. However, as shown in Table S1 of the Supporting Information, these characteristics were not significantly correlated with any of the circulating miRs. Furthermore, in a clinical model circulating miRs were strong predictors of ACS independent of other risk factors including age, hypertension and cholesterol levels (Table 4)

Clinical model with:	OR	95% CI
miR-1	1.30	1.17-1.42
miR-208a	1.16	1.03-1.30
miR-499	1.28	1.18-1.40
miR-21	1.28	1.18-1.39
miR-146a	1.14	1.08-1.21

<u>We emphasized this more in our discussion section (page 9, lines 28-29).</u>

2nd Editorial Decision 16 August 2012

Thank you for the submission of your manuscript "Early assessment of acute coronary syndromes in the emergency department: the potential diagnostic value of circulating microRNAs" to EMBO Molecular Medicine. We have now received the enclosed report from the referee who was asked to re-assess it in light of your previous submission.

As you will see, the reviewer is now supportive of publication and I am glad to let you know that we can proceed with the official acceptance of the manuscript after the following editorial point has been addressed:

- The description of all reported data that includes statistical testing must state the name of the statistical test used to generate error bars and P values, the number (n) of independent experiments underlying each data point (not replicate measures of one sample), and the actual P value for each test (not merely 'significant' or 'P < 0.05'). Please see our Instructions to Authors for more details.

Please submit your revised manuscript within two weeks. I look forward to seeing a revised form of your manuscript as soon as possible.

Yours sincerely,

Editor
EMBO Molecular Medicine

***** Reviewer's comments *****

Referee #1:

In the revised version of the paper it has been received on most of my suggestions for improvement and criticism by the authors. Several questions have been adequately answered which led to a significant improvement of the present manuscript.
In particular, the use of a hsTroponin- Assay as diagnostic standard-marker allows a practical comparison between the use of circulating Mirco-RNAs and clinically established standard-markers.
The findings of the paper are truly spectacular and suggest - at least for the present 'evaluation-cohort' - that especially circulating concentrations of miR-499 provide relevant diagnostic information - in particular for troponin-negative NSTE-ACS-patients in the early stages of disease.
In addition, these results agree with findings of other current studies quite well.
I congratulate the authors on the revised version of this work.

1st Revision - Authors' Response 23 August 2012

We appreciate the comments from the reviewer and the editor. We have changed the description of all reported data that includes statistical testing as requested according to the "Instructions to Authors".
Applied changes to the manuscript were underlined.

Referee #1:
In the revised version of the paper it has been received on most of my suggestions for improvement and criticism by the authors. Several questions have been adequately answered which led to a significant improvement of the present manuscript. In particular, the use of a hsTroponin- Assay as diagnostic standard-marker allows a practical comparison between the use of circulating Mirco-RNAs and clinically established standard-markers. The findings of the paper are truly spectacular and suggest - at least for the present 'evaluation-cohort' - that especially circulating concentrations of miR-499 provide relevant diagnostic information - in particular for troponin-negative NSTE-ACS-patients in the early stages of disease. In addition, these results agree with findings of other current studies quite well. I congratulate the authors on the revised version of this work.

We thank the reviewer for his kind comments and are glad to hear that we adequately answered the criticism raised by the reviewer(s).

Editor:
The description of all reported data that includes statistical testing must state the name of the statistical test used to generate error bars and P values, the number (n) of independent experiments underlying each data point (not replicate measures of one sample), and the actual P value for each test (not merely 'significant' or 'P < 0.05'). Please see our Instructions to Authors for more details.

We reported actual P values as requested, including Figure 1 and Figure 2 and Supporting Information. Furthermore, we added N as well as explanation on error bars and P values when missing (changes were underlined in the manuscript, including both table descriptions and figure legends).
