# Supplementary material for: Early assessment of acute coronary syndromes in the emergency department: the potential diagnostic value of circulating microRNAs
Source: EMBO Mol Med. 2012 Oct 1;4(11):1176–85. doi: 10.1002/emmm.201201749 (PMC3494874; doi:10.1002/emmm.201201749)
Supplement: Supplementary file 2 [file emmm0004-1176-SD2.pdf]

SUPPORTING INFORMATION

Early assessment of acute coronary syndromes in the emergency department: the potential diagnostic value of circulating microRNAs.

Martinus I.F.J. Oerlemans, MD[1], Arend Mosterd, MD, PhD[1,2,3], Marieke S. Dekker MD[2,3,4], Evelyn A. de Vrey, MD[3], Alain van Mil, MSc[1,5], Gerard Pasterkamp, MD, PhD[1], Pieter A. Doevendans, MD, PhD[1,5], Arno W. Hoes, MD, PhD[2], Joost P.G. Sluijter, PhD[1,5]

[1]Department of Cardiology, Division of Heart & Lungs, University Medical Center Utrecht, the Netherlands

[2]Julius Center for Health Sciences and Primary Care, University Medical Center, Utrecht, the Netherlands

[3]Department of Cardiology, Meander Medical Center, Amersfoort, the Netherlands

[4]Department of Cardiology, Isala Clinics, Zwolle, the Netherlands

[5]Interuniversity Cardiology Institute Netherlands (ICIN), Utrecht, the Netherlands

TABLE OF CONTENT

Table S1 - Correlation of patient characteristics with circulating microRNAs in ACS-suspected patients (n=332)

Table S2 - Correlation of antiplatelet or heparin therapy with circulating microRNAs in ACS-suspected patients (n=332)

Table S3 – AUCs and Odds Ratios of miRNAs in suspected ACS patients with symptoms onset <3hours in a clinical model (n=152)

Table S4 - Diagnostic value the combined miRs and myocardial necrosis markers in suspected ACS patients (n=332)

Table S5 - Patient Characteristics of included versus excluded Patients With Chest Pain (n=470)

Figure S1 - Overview of patient inclusion, blood collection and quantification of circulating miRs

Table S1 - Correlation of patient characteristics with circulating microRNAs in ACS-suspected patients (n=332)

Characteristic	miR-1	P	miR-208a	P	miR-499	P	miR-21	P	miR-146a	P
Age	0.154	0.12	-0.026	0.63	0.139	0.07	0.071	0.19	0.013	0.82
Gender	0.032	0.55	-0.063	0.25	0.088	0.10	0.032	0.55	-0.026	0.64
Onset of chest pain	0.001	0.99	0.017	0.76	0.034	0.54	-0.051	0.37	-0.073	0.20
Current smoker	0.078	0.15	-0.026	0.63	0.058	0.29	-0.022	0.69	0.050	0.36
Former smoker	0.071	0.19	-0.080	0.15	0.113	0.07	0.045	0.41	-0.055	0.32
Hypertension	-0.003	0.95	-0.080	0.11	-0.129	0.07	0.026	0.64	-0.012	0.83
Hypercholesterolemia	-0.086	0.12	-0.011	0.85	-0.093	0.09	-0.110	0.09	-0.080	0.14
Diabetes Mellitus	0.004	0.94	-0.009	0.87	-0.114	0.07	-0.071	0.19	-0.019	0.73
Body mass index (kg/m²)	0.013	0.84	-0.013	0.84	-0.019	0.77	0.009	0.89	-0.103	0.11
Family history of CVD	0.124	0.09	0.136	0.07	0.028	0.61	0.011	0.84	-0.077	0.16
History of CVD	0.020	0.72	-0.035	0.52	-0.067	0.21	0.028	0.60	0.004	0.94

Correlation given as Spearman's correlation coefficient (R), CVD = cardiovascular disease;

Table S2 - Correlation antiplatelet or heparin therapy with circulating microRNAs in ACS-suspected patients (n=332)

Therapy	miR-1	P	miR-208a	P	miR-499	P	miR-21	P	miR-146a	P
Aspirin	0.16	0.11	-0.03	0.79	0.08	0.41	-0.18	0.08	-0.10	0.06
Clopidogrel	0.11	0.27	-0.19	0.06	-0.06	0.57	-0,14	0.17	-0.11	0.26
Heparin	0.04	0.69	0.09	0.39	-0.10	0.34	-0.05	0.60	0.03	0.78

Correlation given as Spearman's correlation coefficient (R);

Table S3 – AUCs and Odds Ratios of miRNAs in suspected ACS patients with symptom onset <3hours in a clinical model (n=152)

Marker	AUC	95% CI	OR†	95% CI
Clinical model (CM)	0.78	0.71-0.85	NA	NA
CM + Cardiac troponin	0.88	0.83-0.94	NA	NA
CM + Cardiac hs-troponin T	0.89	0.84-0.94	NA	NA
CM + Cardiac hs-troponin T with:				
miR-1	0.92[a]	0.88-0.96	1.27	1.10-1.47
miR-208a	0.90	0.86-0.95	1.28	1.07-1.54
miR-499	0.92[b]	0.88-0.96	1.32	1.15-1.52
miR-21	0.92[b]	0.88-0.96	1.27	1.13-1.44
miR-146a	0.90	0.85-0.95	1.12	1.00-1.21
miR-1 + miR-499 + miR-21	0.94[b]	0.90-0.98	NA	NA

CM = Clinical model (age, sex, hypertension, hypercholesterolemia, family history, current and former smoking, diabetes mellitus, and history of myocardial infarction, PCI or coronary bypass surgery); AUC = Area AUC = Area under the ROC Curve; 95% CI = 95% Confidence Interval; [a]P=0.004, [b]P<0.001 *versus* hs-troponin T; NA = not applicable; † Adjusted for clinical model and cardiac hs-troponin T.

Table S4 – Diagnostic value the combined miRs and myocardial necrosis markers in suspected ACS patients (n=332)

	All patients (n=332)		Hs-troponin negative patients (n=194)		Patients with symptoms <3 hours (n=152)	
Marker	AUC	95% CI	AUC	95% CI	AUC	95% CI
Myoglobin/CK-MB/hs-troponin T	0.87	0.83-0.92	0.87	0.81-0.94	0.89	0.84-0.95
miR-1/ miR-499/miR-21	0.89	0.86-0.93	0.94	0.90-0.99	0.92	0.88-0.97
P	0.41		0.001			0.07

AUC = Area under the ROC Curve; CK-MB = MB fracion of creatine kinase; 95% CI = 95% Confidence Interval; P value *versus* myoglobin/CK-MB/hs-troponin T.

Table S5 – Patient Characteristics of included *versus* excluded Patients With Chest Pain

Characteristic	Excluded (n=138)	Included (n=332)	P
N (%)	138 (29.4)	332 (70.6)	
Age (yrs)	61.7±14.9	62.9±14.4	0.42
Male sex (% male)	73 (53.5)	190 (57.2)	0.44
Onset of chest pain, median (IQR)	3.2 (2.0-6.7)	3.2 (1.8-8.0)	0.89
Risk factors			
Current smoker	39 (28.2%)	86 (26.1)	0.45
Former smoker	34 (24.6%)	103 (31.5)	0.11
Hypertension	57 (41.3)	151 (45.8)	0.25
Hypercholesterolemia	42 (30.4%)	109 (33.0)	0.42
Diabetes Mellitus	22 (15.9%)	52 (15.8)	0.90
Body mass index (kg/m²)	26.7±4.8	26.3±5.8	0.69
Parental CVD	50 (36.2%)	133 (40.7)	0.24
History of CVD	67 (48.6%)	159 (47.9)	0.91
Cardiac Troponin I (µg/L)	0.03 (0.02-0.04)	0.03 (0.02-0.05)	0.83
Cardiac hs-Troponin T (pg/mL)	5.3 (2.8-18.2)	6.0 (2.2-18.7)	0.77

Data presented as mean±SD for age, body mass index and as median (25th – 75th interquartile range) for onset of chest pain and cardiac troponin levels. All other variables are presented as n (%); ACS = acute coronary syndrome; CVD = cardiovascular disease; P value excluded *versus* included patients with chest pain, based on RNA quality. Mann-Whitney (continuous variables) or χ^2 test (categorical variables).

Supporting Information Fig. S1

Overview of patient inclusion, blood collection and quantification of circulating miRs

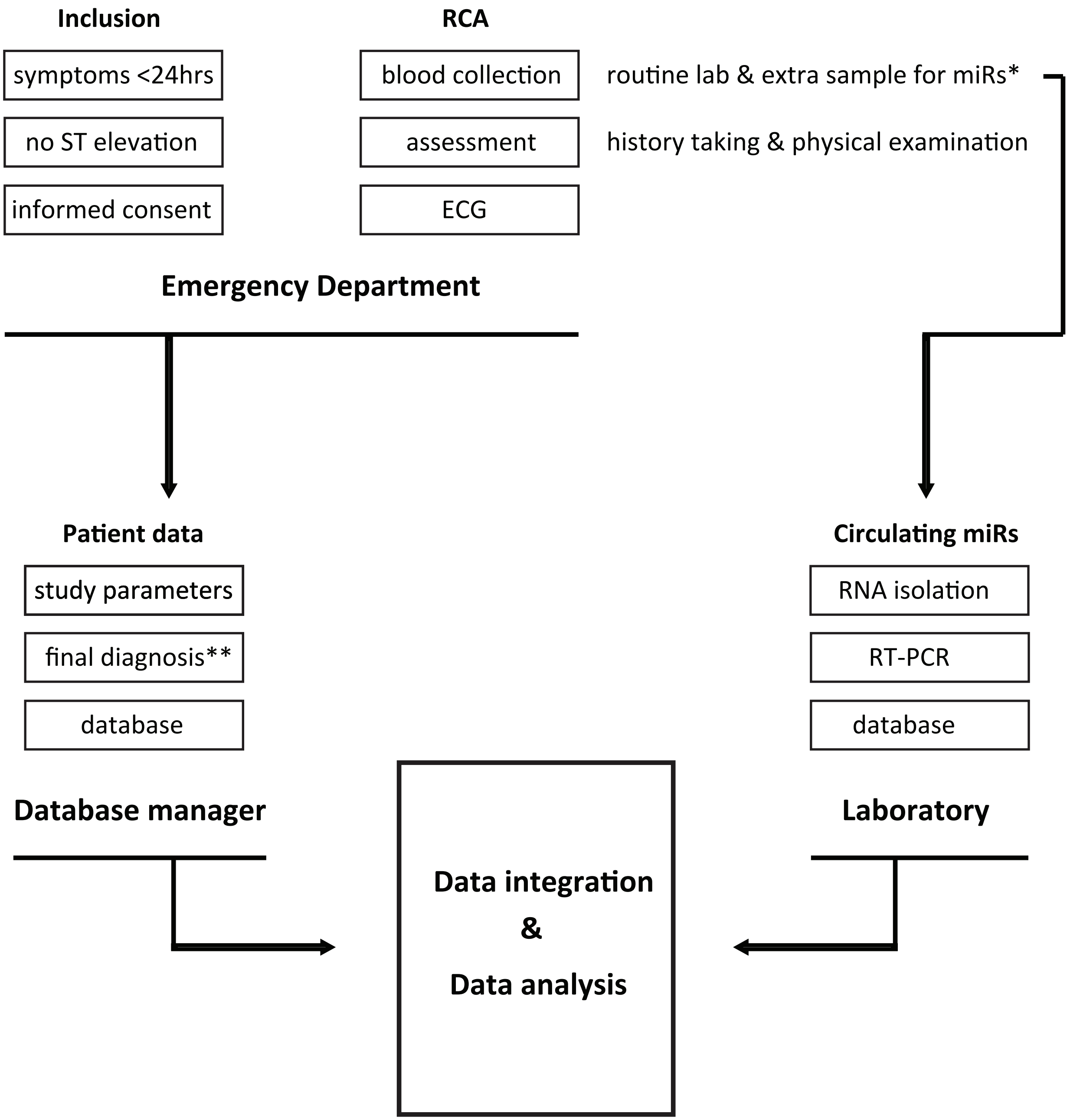

RCA = routine clinical assessment

*collected at presentation; **by expert panel based on all available clinical data
